# Establishment of A Protocol for *In Vitro* Culture of Cardiogenic Mesodermal Cells Derived from Human Embryonic Stem Cells

**DOI:** 10.22074/cellj.2019.5661

**Published:** 2018-08-07

**Authors:** Sadaf Vahdat, Sara Pahlavan, Nasser Aghdami, Behnaz Bakhshandeh, Hossein Baharvand

**Affiliations:** 1 Department of Biotechnology, College of Science, University of Tehran, Tehran, Iran; 2 Department of Stem Cells and Developmental Biology, Cell Science Research Center, Royan Institute for Stem Cell Biology and Technology, ACECR, Tehran, Iran; 3 Department of Developmental Biology, University of Science and Culture, Tehran, Iran

**Keywords:** Cardiomyocytes, Cell Differentiation, Matrigel, Multipotent Stem Cells

## Abstract

**Objective:**

Cardiovascular progenitor cells (CPCs) are introduced as one of the promising cell sources for preclinical studies and regenerative medicine. One of the earliest type of CPCs is cardiogenic mesoderm cells (CMCs), which have the capability to generate all types of cardiac lineage derivatives. In order to benefit from CMCs, development of an efficient culture strategy is required. We aim to explore an optimized culture condition that uses human embryonic stem cell (hESC)-derived CMCs.

**Materials and Methods:**

In this experimental study, hESCs were expanded and induced toward cardiac lineage in a suspension culture. Mesoderm posterior 1-positive (MESP1^+^) CMCs were subjected to four different culture conditions: i. Suspension culture of CMC spheroids, ii. Adherent culture of CMC spheroids, iii. Adherent culture of single CMCs using gelatin, and iv. Adherent culture of single CMCs using Matrigel.

**Results:**

Although, we observed no substantial changes in the percentage of MESP1^+^ cells in different culture conditions, there were significantly higher viability and total cell numbers in CMCs cultured on Matrigel (condition iv) compared to the other groups. CMCs cultivated on Matrigel maintained their progenitor cell signature, which included the tendency for cardiogenic differentiation.

**Conclusion:**

These results showed the efficacy of an adherent culture on Matrigel for hESC-derived CMCs, which would facilitate their use for future applications.

## Introduction

Cardiovascular progenitor cells (CPCs) are proliferative multipotent cardiac-committed cells that can generate all main types of cardiac cells (cardiomyocytes, endothelial and smooth muscle cells) *in vitro* and *in vivo* (1). They are widely used in various experimental and clinical studies. CPCs are considered superior candidates for cardiac cell therapy due to their cardiac regeneration capacity where they have the capability to replace dead myocardium as well as exert paracrine effects (2-4). These progenitor cells can be isolated from the biopsy of a patient’s heart, expanded *in vitro*, and transplanted back to the heart as autologous cells (5). 

However, increased age affects the functionality and proliferative capacity of patient-derived CPCs (5). Today, CPCs can be differentiated from all sources of human pluripotent stem cells (hPSCs) such as human embryonic stem cells (hESCs) and human induced pluripotent stem cells (hiPSCs). CPCs are considered an alternative and readily available source for experimental and clinical applications (6-9). There are a number of well-established protocols that differentiate hPSCs toward cardiac lineages by manipulating signaling pathways involved in cardiogenesis. Therefore, hPSCs can be used for generation and specification of CPCs (10-12). 

hPSC-derived CPCs can successfully differentiate into all 3 types of cardiac lineages *in vitro* and could improve cardiac function after transplantation into animal models of myocardial infarction (1315). All CPC types arise from a common ancestor progenitor cell, which is featured by the expression of mesoderm posterior 1 (*MESP1*) transcription factor. *MESP1* expression is specific to the early stage of heart development and considered to be the master regulator of cardiac development. Therefore, it is an appropriate marker for isolation of early CPCs, or cardiogenic mesoderm cells (CMCs) (16-18). Despite the importance of *MESP1*^+^ CMCs in normal heart development and their potential application *in vitro* as well as clinical preparations (19-21), no optimum condition exists for their culture. Therefore, development of an efficient culture condition that can retain cellular features and provide the possibility of further manipulations are inevitably required.

In this study, we aimed to establish an efficient culture condition for hESC-derived CMCs. CMCs were more than 80% positive for *MESP1* and expressed cardiac transcription factors. Their differentiation potency toward cardiomyocytes were preserved as shown by induction of both spontaneous and directed differentiation.

## Materials and Methods

### Expansion of human embryonic stem cells in suspension culture

In this experimental study, hESCs (RH5 line) were cultured and expanded as spheroids according to a previously described protocol (22). Briefly, 2×10^5^ viable cells/ml were cultured in hESC medium that consisted of Dulbecco’s Modified Eagle Medium/ Ham’s F-12 (DMEM/F12, Gibco, USA) supplemented with 20% knockout serum replacement (KOSR, Gibco, USA), 1% insulin-transferrin-selenite (Gibco, USA), 1% nonessential amino-acids (NEAA, Gibco, USA), 1% penicillin/streptomycin (Gibco, USA), 0.1 mM ß-mercaptoethanol (Sigma-Aldrich, USA), and 100 ng/ ml basic fibroblast growth factor (bFGF, Royan Biotech, Iran) in non-adhesive bacterial plates. The medium was renewed every 2 days. When spheroids reached 200-250 µm, they were dissociated into single cells with Accutase solution (Sigma-Aldrich, USA), and replated on new bacterial plates at a 1:3 ratio. Cells were treated with 10 µM of ROCK inhibitor (ROCKi, Sigma-Aldrich, USA) for the first 2 days. 

### Directed differentiation of human embryonic stem cells into cardiogenic mesoderm cells

hESC spheroids (175-200 µm in diameter) were subjected to directed differentiation into CMCs as previously described (23). Briefly, spheroids were cultured in basal differentiation medium that contained RPMI 1640 (Gibco, USA) supplemented with 2% B-27 (Gibco, USA), 2 mM L-glutamine (Gibco, USA), 1% penicillin/streptomycin, 1% NEAA, 0.1 mM ß-mtercaptoethanol, and 12 µM of small molecule (SM) CHIR99021 (Stemgent, USA) for 24 h followed by 24 h culture in basal differentiation media without CHIR99021.

### Cardiogenic mesoderm cell culture conditions

To optimize culture of hESC-derived CMCs, we collected CMC spheroids on day 2 post-differentiation and cultured these spheroids in 4 different culture conditions: i. Suspension culture of CMC spheroids, ii. Adherent culture of CMC spheroids on gelatin, iii. Adherent culture of single CMCs on gelatin, and iv. Adherent culture of single CMCs on Matrigel.

i. In the first approach, we cultured the spheroids of hESC-derived CMCs in a suspension culture condition with non-adhesive bacterial plates. ii. The second culture condition was designed to plate CMC spheroids on gelatin-coated tissue culture dishes to enable them to grow and adhere. The last protocol included enzymatic dissociation of CMC spheroids followed by plating single CMCs on tissue culture dishes to enable them to grow and adhere to the dishes. Briefly, CMC spheroids were treated with Accutase solution for 3 minutes at 37°C and centrifuged at 1500 rpm for 5 minutes. The resultant individual CMCs were cultured on 0.1% gelatin (condition iii) or Matrigel-coated tissue culture plates (condition iv) at a cell density of 10^5^ cells/cm^2^. Cells were treated overnight with 10 µM ROCKi. The media was refreshed every 2 days for all groups by SM-free differentiation medium.

### Flow cytometry and immunostaining

On day 2, RH5 spheroids were dissociated into single cells by using Accutase solution, washed with phosphate-buffered saline (PBS)/0.5% w/v bovine serum albumin (BSA, Sigma-Aldrich, USA), and fixed with 1% paraformaldehyde for 20 minutes at room temperature (RT). Following another wash, the cells were treated with ice-cold 90% methanol (Merck, USA) at 4°C for 15 minutes, washed twice with PBS/0.5% BSA, and incubated overnight with primary antibody *MESP1* (Abcam, USA) in PBS/0.5% BSA/0.1% Triton X100 (Sigma-Aldrich, USA) at 4°C. The next day, cells were washed and incubated with Alexa488-conjugated donkey anti-mouse secondary antibody (Invitrogen, USA) in PBS/0.5% BSA/0.1% Triton X100 for 1 hour at RT. Cells were analyzed by a BD FACSCalibur (BD Biosciences, San Jose, CA, USA) system. The data was analyzed by Flowing Software 2.5 (Turku Centre for Biotechnology, Finland).

Immunofluorescent staining of the cells was conducted by plating them on Matrigel-coated plates for 24 hours. The cells were fixed with 1% paraformaldehyde for 20 minutes at RT. In the subsequent steps, we used the same protocol as flow cytometry. Cells were stained with DAPI as a counterstain and observed by a fluorescent microscope (Olympus, Japan). *MESP1*, Ki67 (Abcam, USA), MHC (Abcam, USA), cTNT (Abcam, USA), and SMA (Abcam, USA) were the primary antibodies. Alexa488-conjugated donkey anti-mouse (Invitrogen, USA), Alexa546-conjugated donkey anti-rabbit (Invitrogen, USA), and Alexa546conjugated donkey anti-goat (Invitrogen, USA) were the secondary antibodies. We quantified the positively stained cells by randomly selecting 4 fields for each marker. The number of positive cells were divided by the total cells of each field (stained with DAPI).

### Gene transcription assessment

Total RNA was manually isolated as previously described (5). First strand cDNA synthesis was performed using a PrimeScript^TM^ RT Reagent Kit (Perfect Real Time) (Takara, Japan) and quantitative PCR was done using a SYBR Premix Ex Taq Kit (Takara, Japan) with a Rotor Gene Corbett System (R080873). The 2^-ΔΔct^ formula was used to calculate relative gene expression of cells at day 2 compared to RH5 undifferentiated cells at day 0. *GAPDH* was the housekeeping gene. All primers’ information is summarized in Table 1. 

**Table 1:** Primer sequences used for quantitative real time reverse-transcription polymerase chain reaction (RT-PCR)


Gene name	Primer sequences (5´-3´)

*MESP1*	F: ACCTTCGAAGTGGTTCCTTG
	R: TCCTGCTTGCCTCAAAGTGT
*ISL1*	F: TACAAAGTTACCAGCCACC
	R: GGAAGTTGAGAGGACATTGA
*PDGFRA*	F: TACACTTGCTATTACAACCACA
	R: ATCCTCCACGATGACTAAAT
*KDR*	F: CCAGCCAAGCTGTCTCAGT
	R: CTGCATGTCAGGTTGCAAAG
*NKX2.5*	F: TCTATCCACGTGCCTACAG
	R: CCTCTGTCTTCTCCAGCTC
*MEF2c*	F: TCCGAGTTCTTATTCCACC
	R: ATCCTCCCATTCCTTGTC


### Viability and expansion assessment of human embryonic stem cell-derived cardiogenic mesoderm cells 

CMCs were dissociated into single cells with the Accutase solution at 37°C for 3 minutes. The enzyme was removed by centrifuging the cell suspension at 1500 rpm for 5 minutes. The resultant cell pellet was dissolved in 5 ml of medium. We mixed 50 µl of the cell suspension with 50 µl of 0.4% trypan blue and loaded 10 µl of the pipetted mixture into a hemocytometer. The cell count was done with a ×10 microscope lens and we calculated the viability of each sample as the ratio of viable cells (without color) to all counted cells. We measured the fold change of expansion after cultivation by dividing the cell count on day 5 to the seeding count. 

### Spontaneous and directed cardiogenic differentiation of cardiogenic mesoderm cells

We evaluated the cardiac differentiation potential of the cultured CMCs for both spontaneous and directed differentiations. For spontaneous differentiation, the cultured cells grew for 20 days in basal differentiation medium without any additional SM treatment. Directed differentiation was induced by treatment of the cultured cells with a cardiogenic cocktail that included 5 µM IWP2 (Tocris, England), 5 µM purmorphamine (Stemgent, USA), and 5 µM SB431542 (Cayman, USA) for 2 days. The medium was refreshed every 3 days.

### Electrophysiological study of differentiated cardiogenic mesoderm cells

Functional studies were performed by obtaining the field potential recording according to a previously described method (23). Briefly, the selected beating clusters were mechanically detached under a stereo microscope (Olympus, Japan). Each beating cluster was then plated on a Matrigel-coated multielectrode array (MEA) plate and cultured overnight. On the day of the experiment, we connected the plates to a head stage amplifier to record the field potentials at a sampling rate of 2 kHz.

### Statistical analysis

All datasets were obtained from 3 independent biological replicates and presented as mean ± standard deviation (SD). Statistical analysis was performed with SPSS 16.0 (SPSS Inc., USA) according to unpaired t test or one-way ANOVA with Tukey’s post-hoc, depending on the results of the normality test. P=0.05 was considered statistically significant.

## Results

### Characterization of human embryonic stem cell- derived cardiogenic mesoderm cells

**Fig.1:**
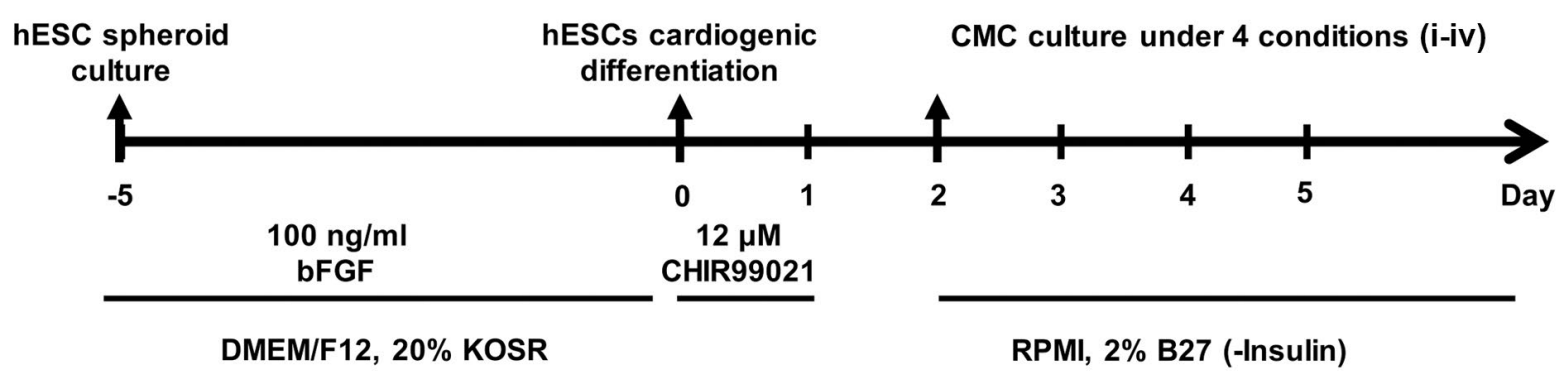
Schematic diagram of the strategy used to establish a suitable culture condition for human embryonic stem cell (hESC)-derived cardiogenic mesoderm cells (CMCs). hESCs were expanded and differentiated into cardiomyocytes in suspension culture. One day after treatment of hESC spheroids with small molecule (SM) CHIR99021 (day 2), we obtained the highest percentage of *MESP1*^+^ CMCs. These cells were subjected to 4 different culture conditions in medium without small molecules: i. Suspension culture of CMC spheroids, ii. Adherent culture of CMC spheroids on gelatin, iii. Adherent culture of single CMCs on gelatin, and iv. Adherent culture of single CMCs on Matrigel.

**Fig.2:**
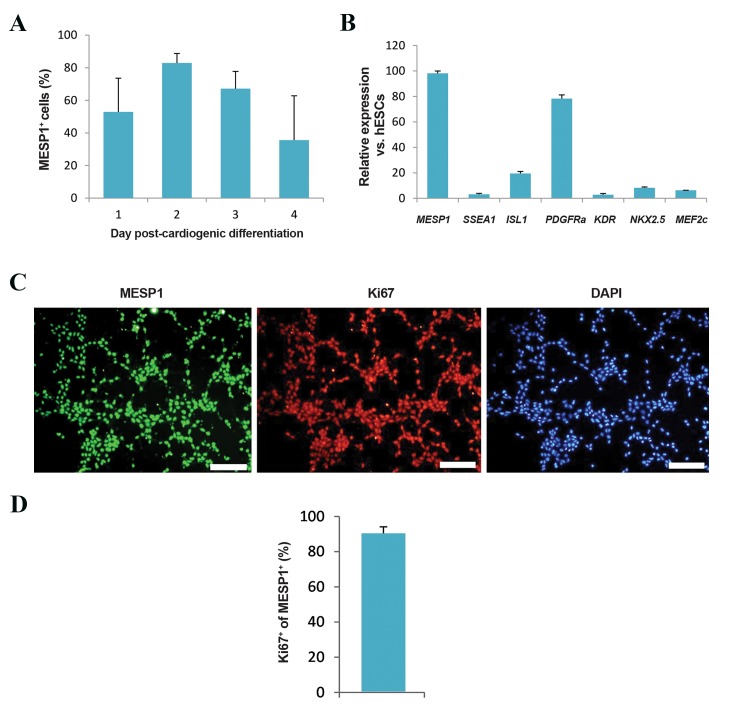
Characterization of human embryonic stem cell (hESC)-derived cardiogenic mesoderm cells (CMCs). A. Flow cytometry analysis of differentiated hESC spheroids on days 1 to 4 after differentiation for *MESP1*. We observed the highest percentage of *MESP1*^+^ cells 2 days after differentiation, B. Gene expression profile of CMCs. Cardiac transcription factors (*ISL1, NKX2.5,* and *MEF2c*), CMC surface markers (*KDR, PDGFRa,* and *SSEA1*) and *MESP1* were upregulated compared to undifferentiated hESCs, and C, D. Immunostaining of CMCs. More than 90% of the *MESP1*^+^ cells were positive for Ki67 (scale bar: 200 µm).

In order to generate hESC-derived CMCs, we subjected the hESCs to cardiogenic differentiation as described previously (Fig .1) (23). *MESP1* expression was evaluated during the first 4 days post-differentiation. Flow cytometry analysis showed the highest percentage of *MESP1*^+^ cells (82.8 ± 5.9%) at day 2 after cardiogenic differentiation (Fig .2A). Therefore, we selected CMCs from this time point for the remainder of the experiments. 

CMCs were further characterized by evaluation of expressions of cardiac commitment transcription factors (*ISL1, NKX2.5,* and *MEF2c*) and CMC markers (*MESP1, KDR, PDGFRa,* and *SSEA1*). In addition to substantial upregulation of *MESP1* and *PDGFRa,* CMCs showed an increase in expression of the CPC transcription factors on day 2 compared to undifferentiated hESCs (Fig .2B). They also expressed *SSEA1,* a well-known surface marker for CMCs (11, 14, 24). Immunostaining showed that more than 90% of *MESP1*^+^ CMCs were Ki^67+^ (Fig .2C, D). 

### *In vitro* culture of human embryonic stem cell-derived cardiogenic mesoderm cells 

**Fig.3:**
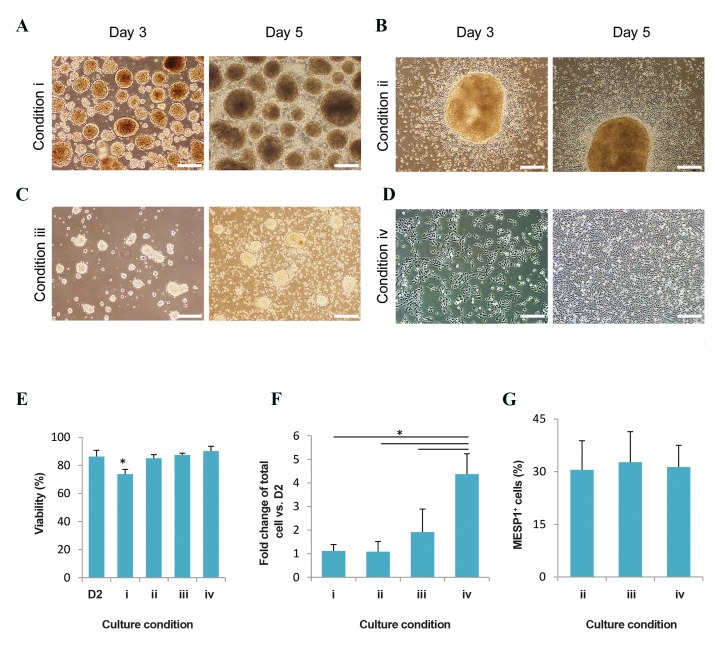
Cultivation conditions for human embryonic stem cell (hESC)-derived cardiogenic mesoderm cells (CMCs). After generation of suspended CMCs as spheroids, we cultured these spheroids for 3 days under 4 conditions. A. Culture of intact spheroids in non-adhesive bacterial plate (condition i), B. Replating of intact spheroids on gelatin-coated plate (condition ii), C. Replating of dissociated spheroids on gelatin-coated plate (condition iii), D. Replating of dissociated spheroids on Matrigel-coated plate (condition iv) (scale bar: 200 µm for all images), E. Viability assessment of cultured CMCs 3 days after culture in the 4 different culture conditions (day 5). CMC spheroids in suspension culture (condition i) showed significant reduction in cell viability (~15%) at day 5 compared to day 2 (*; P≤0.05), F. Expansion capacity of CMCs at day 5 in the 4 different culture conditions. The ratio of output cells to seeding cells was significantly higher in CMCs cultured on Matrigel (condition iv) compared to the other 3 approaches (*; P≤0.05), and G. Flow cytometry analysis of *MESP1*^+^ cells. There were no significant differences between culture conditions based on the percentage of *MESP1*^+^ cells. D2; Day 2.

We sought to find the optimal culture condition for hESC-derived CMCs. Differentiated spheroids at the cardiogenic mesoderm stage were cultured for 3 days under 4 conditions: i. Culture of intact spheroids in nonadhesive bacterial plate, ii. Replating of intact spheroids on gelatin-coated plate, iii. Replating of dissociated spheroids on gelatin-coated plate, and iv. Replating of dissociated spheroids on Matrigel-coated plate (Fig .3A-D). Condition i had decreased viability after 3 days of suspension culture (Fig .3E). However, the cell viability did not change in the other conditions (Fig .3E); therefore, we removed condition i for the rest of the experiments. The culture of dissociated spheroids on Matrigel resulted in higher numbers of CMCs (more than 4-fold) compared to the other conditions (Fig .3F). However, the percentage of *MESP1*^+^ cells did not significantly differ between conditions ii-iv (Fig .3G). Based on the above results, we chose the Matrigel-based adherent culture as an efficient culture condition for the rest of the experiments. 

### Cardiogenic differentiation of human embryonic stem cell-derived cardiogenic mesoderm cells

**Fig.4:**
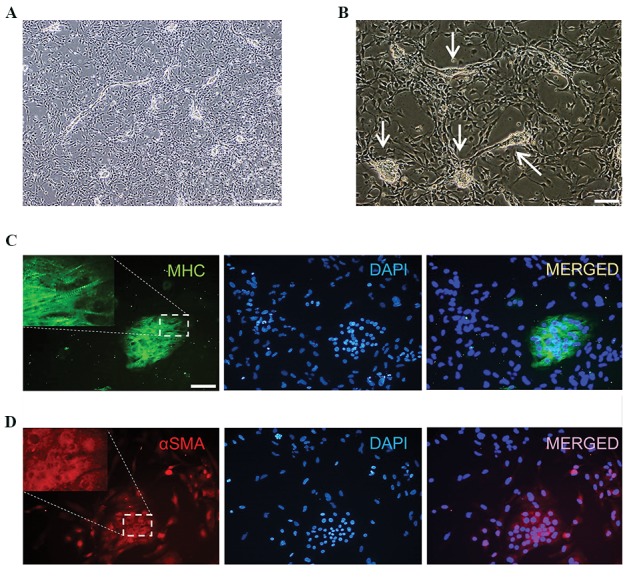
Spontaneous differentiation potential of cultured cardiogenic mesoderm cells (CMCs) on Matrigel. A. Beating clusters generated 12 days after culture of CMCs (scale bar: 200 µm), B. Higher magnification of beating clusters (arrows) (scale bar: 100 µm), C. MHC, and D. a-SMA staining of differentiated CMCs. Cells were counterstained with DAPI (scale bar: 100 µm).

We sought to determine if cultured CMCs could maintain their differentiation potency. The CMCs were subjected to both spontaneous and directed differentiation. For spontaneous differentiation, CMCs were kept in culture for an additional 20 days without any special treatment. After 10 days, the CMCs began to generate some clusters. We observed the first beating clusters on day 14 (Fig .4A, B). Spontaneously differentiated CMCs were positive for MHC and a-SMA as analyzed by immunostaining, which indicated the differentiation potential of CMCs into a cardiac lineage (Fig .4C, D). 

**Fig.5:**
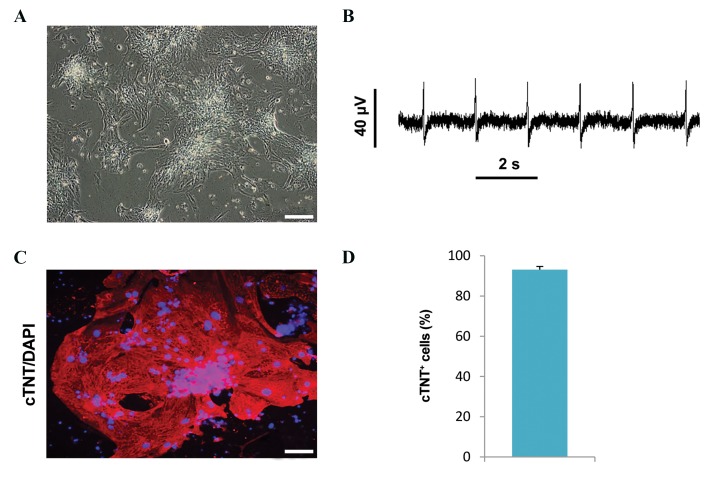
Directed differentiation of human embryonic stem cell (hESC)-derived cardiogenic mesoderm cells (CMCs). A. Morphology of beating clusters generated by directed differentiation of CMCs (scale bar: 200 μm), B. Representative field potentials recorded from differentiated CMCs, and C, D. Immunostaining of a cardiomyocyte structural marker (cTNT). More than 90% of cells were cTNT^+^ (C: scale bar: 100 μm).

In order to direct the CMCs differentiation into cardiomyocytes, we subjected the CMCs to a cardiogenic cocktail (IWP2, purmorphamine, and SB431542). Cells began to beat on day 7 ± 1 post-treatment. The number of beating clusters increased until 100% beating occurred on day 12 ± 2 (Fig .5A). Beating clusters were replated on Matrigel-coated MEA plates on day 30 in order to evaluate their electrophysiological properties. Directed differentiation of CMCs resulted in rhythmic field potentials (Fig .5B). Immunostaining of the cardiac cytoskeletal marker, cTNT, showed a high percentage of cTNT+ cells (93.1 ± 1.6%) in CMC- derived cardiomyocytes, which indicated the well- preserved differentiation capacity of cultured CMCs (Fig .5C, D). 

## Discussion

hPSCs possess special characteristics such as unlimited self-renewal and differentiation potential, which make them suitable tools for human regenerative medicine. They have been widely used in experimental setups, developmental studies and clinical oriented research. In the cardiovascular field, the generation and culture of hPSC-derived cardiac lineage cells received high attention due to their potential use in cell therapies (11, 25, 26). hPSC-derived cardiovascular cells can be used for developmental research, genetic manipulation, drug screening, and tissue engineering (9, 12, 27, 28). Therefore, a suitable culture condition that could preserve the cellular characteristics of CMCs or CPCs is highly required.

In this study, we attempted to find a culture condition for *MESP1*^+^ CMCs, one of the earliest CPCs during heart development (20, 29-32). In line with our previous report, we identified the highest population of *MESP1*^+^ CMCs on day 2 post-differentiation, which was immediately before cell treatment with the cardiogenic cocktail. *MESP1* expression began on day 1, peaked on day 2, and downregulated after cardiogenic induction (23). The gene expression profile of *MESP1*^+^ CMCs showed expression of cardiac transcription factors ISL1, NKX2.5, and MEF2c as well as CMC markers *MESP1* and PDGFRa which exhibited a typical pattern of early CPCs (14, 20, 26, 33). The CMCs were positive for Ki67, which showed their proliferative state. CMC spheroids were used to find the best culture strategy that had the most expansion capacity. These CMC spheroids were enriched for more than 80% *MESP1*^+^ cells; therefore, there was no need for additional cell purification with sorting systems (34). 

In contrast to published protocols that used gelatin as a culture substrate for *ISL1^+^/MEF2c^+^* and *Nkx2.5^+^* CPCs (25, 35), the *MESP1*^+^ CMCs in the current study greatly attached to, spread, and grew on Matrigel. Matrigel is a well-known substrate for several types of stem cells, including hPSCs (36). Of note, different cell types have different attachment properties, which highlights the importance of finding a suitable culture substrate for each cell type (37). 

Based on our results, the Matrigel-based adherent culture of single CMCs was identified as an efficient culture condition among the 4 different tested culture conditions. We did not use any maintenance medium for the CMCs culture; therefore, the percentage of *MESP1*^+^ cells decreased after 3 days in culture, which might show the initiation of spontaneous differentiation. However, to further evaluate the differentiation potency of cultured CMCs, we subjected them to spontaneous and directed differentiation. Spontaneously differentiated cells generated cardiomyocytes as well as smooth muscle cells that stained for MHC and SMA, respectively. However, after directed differentiation, approximately 93% of cells were positive for cTNT, which showed their

high capacity for differentiation into cardiomyocytes, consistent with our previous report (23). During heart development, *MESP1* cooperates its paralogous gene *MESP2* to initiate cardiogenesis based on the neighboring signals. Additionally, *MESP1* expression is essential for epithelial-mesenchymal transition (EMT) which promotes the migration of CPCs from the primitive streak (19, 29).

Matrigel can affect cell morphology and differentiation capacity due to its composition (38). Additionally, Matrigel can retain cell properties, which is in line with our results. The Matrigel-based adherent culture of CMCs might provide a suitable condition for application of genetic tools such as siRNA gene knockdown as well as small molecule/drug screenings (35).

## Conclusion

We attempted to find a suitable culture condition for hESC-derived *MESP1*^+^ cells. Matrigel-based adherent culture of CMCs could well preserve their characteristics that included proliferation and differentiation capacity into cardiac lineages, which would facilitate their application for further cell manipulations.
